# Changes in reporting for unintentional injury deaths, United States of America

**DOI:** 10.2471/BLT.18.215327

**Published:** 2019-01-18

**Authors:** Peishan Ning, David C Schwebel, Haitao Chu, Motao Zhu, Guoqing Hu

**Affiliations:** a Xiangya School of Public Health, Central South University, Changsha, China.; b Department of Psychology, University of Alabama at Birmingham, Birmingham, United States of America (USA).; c School of Public Health, University of Minnesota, Minneapolis, USA.; d The Research Institute at Nationwide Children’s Hospital; Ohio State University, Columbus, USA.; e Department of Epidemiology and Health Statistics, Xiangya School of Public Health, Central South University, 110 Xiangya Road, Changsha, Hunan, China.

## Abstract

**Objective:**

To quantify how changes in reporting of specific causes of death and of selecting underlying cause from among multiple causes of death contribute to trends in mortality from unintentional injury in Americans aged 65 years or older.

**Methods:**

We extracted age-standardized unintentional injury mortality data in the United States Centers for Disease Control and Prevention online databases from 1999 to 2016. We used an attribution method to calculate two indicators: the proportion of mortality with specific codes out of all mortality; and the proportion of mortality with underlying cause of death selected from multiple causes of death. We conducted a linear regression to examine the changes over time in these proportions and in reported and age-adjusted mortality.

**Findings:**

From 1999 through 2016, the proportion of cause-specific unintentional injury mortality in this age group increased from 74% in 1999 (136.9 out of 185.0 per 100 000 population) to 85% in 2016 (143.0 out of 169.1 per 100 000 population) based on multiple causes of death codes. The proportions of mortality with underlying cause of death selected out of multiple causes of death rose in all specific causes of unintentional injury except motor vehicle crash. Age-standardized mortality attributed to reporting changes increased steadily between 1999 and 2016. The increases for overall unintentional injury, fall, motor vehicle crash, suffocation, poisoning and fire or hot object were 24.2, 13.5, 2.1, 2.3, 1.6 and 0.4 deaths per 100 000 persons, respectively.

**Conclusion:**

Changes in data reporting affect trends in overall and specific unintentional injury mortality over time for older Americans.

## Résumé

**Objectif:**

Quantifier la manière dont les changements dans l'enregistrement des causes spécifiques des décès et dans le choix de la cause sous-jacente parmi des causes multiples de décès contribuent aux tendances observées en matière de mortalité par traumatisme accidentel chez les Américains de 65 ans et plus.

**Méthodes:**

En utilisant les bases de données en ligne des Centres pour le contrôle et la prévention des maladies aux États-Unis (*Centers for Disease Control and Prevention*), nous avons extrait les données de mortalité par traumatisme accidentel, standardisées selon l'âge, pour la période comprise entre 1999 et 2016. Nous avons utilisé une méthode d'attribution pour calculer deux indicateurs: la proportion de la mortalité associée à des codes spécifiques par rapport à la mortalité totale et la proportion de la mortalité associée à une cause sous-jacente de décès choisie parmi de multiples causes de décès. Nous avons réalisé une régression linéaire pour examiner les changements au fil du temps de ces proportions ainsi que de la mortalité enregistrée et de la mortalité ajustée en fonction de l'âge.

**Résultats:**

Entre 1999 et 2016, la proportion de la mortalité par traumatisme accidentel associée à une cause spécifique a augmenté dans cette tranche d'âges, en passant de 74% en 1999 (136,9 cas sur 185,0 pour 100 000 habitants) à 85% en 2016 (143,0 cas sur 169,1 pour 100 000 habitants) sur la base des codes de causes multiples de décès. Les proportions de la mortalité associée à une cause sous-jacente de décès choisie parmi des causes multiples de décès a augmenté pour toutes les causes spécifiques de traumatismes accidentels, à l'exception des accidents impliquant des véhicules à moteur. La mortalité standardisée selon l'âge attribuée aux changements dans l'enregistrement des décès a augmenté de façon continue entre 1999 et 2016. Les augmentations pour: tous les types de traumatismes accidentels; les chutes; les accidents impliquant un véhicule à moteur; les étouffements; les intoxications et les expositions à des incendies ou à des substances brulantes ont été respectivement de 24,2; 13,5; 2,1; 2,3; 1,6 et 0,4 décès pour 100 000 habitants.

**Conclusion:**

Les changements dans l'enregistrement des données affectent les tendances observées en matière de mortalité générale par traumatisme accidentel et de mortalité par traumatisme accidentel spécifique, au fil du temps, chez les Américains les plus âgés.

## Resumen

**Objetivo:**

Cuantificar cómo los cambios en el reporte de las causas específicas de muerte y de la selección de la causa subyacente de entre las múltiples causas de muerte contribuyen a las tendencias en la mortalidad por lesiones no intencionales en los estadounidenses de 65 años de edad o más.

**Métodos:**

Se extrajeron datos de mortalidad por lesiones no intencionales estandarizados por edad de las bases de datos en línea de los Centros para el Control y la Prevención de Enfermedades de los Estados Unidos desde 1999 hasta 2016. Se utilizó un método de atribución para calcular dos indicadores: la proporción de mortalidad con códigos específicos de entre toda la mortalidad; y la proporción de mortalidad con causa subyacente de muerte seleccionada entre múltiples causas de muerte. Se realizó una regresión lineal para examinar los cambios a lo largo del tiempo en estas proporciones y en la mortalidad informada y ajustada por edad.

**Resultados:**

De 1999 a 2016, la proporción de mortalidad por lesiones no intencionales por causas específicas en este grupo de edad aumentó del 74 % en 1999 (136,9 de 185,0 por 100.000 habitantes) al 85 % en 2016 (143,0 de 169,1 por 100.000 habitantes) sobre la base de los múltiples códigos de defunción. Las proporciones de mortalidad con causa subyacente de muerte seleccionada de entre las múltiples causas de muerte aumentaron en todas las causas específicas de lesiones involuntarias, excepto en los accidentes automovilísticos. La mortalidad normalizada por edad atribuida a los reportes sobre los cambios aumentó constantemente entre 1999 y 2016. Los aumentos por lesiones involuntarias generales, caídas, choques de vehículos de motor, asfixia, envenenamiento e incendios u objetos calientes fueron de 24,2, 13,5, 2,1, 2,3, 1,6 y 0,4 muertes por cada 100.000 personas, respectivamente.

**Conclusión:**

Los cambios en el reporte de datos afectan las tendencias en la mortalidad general y específica por lesiones no intencionales a lo largo del tiempo para los estadounidenses mayores.

## ملخص

**الغرض:**

توضيح كيف أن التغيرات في الإبلاغ عن الوفاة محددة الأسباب، وفي اختيار السبب الكامن من بين عدة أسباب للوفاة، يسهم في اتجاهات الوفيات الناجمة عن الإصابات غير المتعمدة في الأمريكيين الذين تبلغ أعمارهم 65 سنة أو أكثر.

**الطريقة:**

قمنا باستخلاص بيانات الوفيات الناتجة عن الإصابات غير المتعمدة، والقائمة على معيار السن، في قواعد بيانات الإنترنت الخاصة بمراكز مكافحة الأمراض والوقاية منها في الولايات المتحدة الأمريكية من عام 1999 إلى عام 2016. واستخدمنا طريقة الإسناد لحساب اثنين من المؤشرات: نسبة الوفيات مع رموز محددة من جميع الوفيات؛ ونسبة الوفيات مع السبب الأساسي للوفاة المختار من عدة أسباب للوفاة. لقد أجرينا تحوفاً خطيًا لفحص التغيرات بمرور الوقت في هذه النسب وفي معدل الوفيات المُبلّغ عنه والقائم على السن.

**النتائج:**

من عام 1999 حتى عام 2016، ارتفعت نسبة الوفيات الناجمة عن الإصابات غير المتعمدة في هذه الفئة العمرية من 74٪ في عام 1999 (136.9 من 185.0 لكل 100000 نسمة) إلى 85٪ في عام 2016 (143.0 من 169.1 لكل 100000 نسمة) بناء على أسباب متعددة من رموز الموت. ارتفعت نسب الوفيات ذات الأسباب الكامنة للوفاة والمختارة من عدة أسباب للوفاة، في جميع الأسباب المحددة للإصابة غير المتعمدة باستثناء حوادث المركبات. ارتفع معدل الوفيات القائمة على معيار السن، والمنسوبة للتغيرات في الإبلاغ، بشكل مطرد بين عامي 1999 و2016. وكانت الزيادات في إجمالي حالات الإصابات غير المتعمدة، أو السقوط، أو حوادث المركبات، أو الاختناق، أو التسمم والحريق، أو الأشياء الساخنة: 24.2 و13.5 و2.1 و2.3 و1.6 و0.4 حالة وفاة لكل 100000 شخص، على التوالي.

**الاستنتاج:**

تؤثر التغيرات في الإبلاغ على البيانات في اتجاهات وفيات الإصابات غير المتعمدة بشكل عام ومحدد مع مرور الوقت بالنسبة للأمريكان المسنين. ****

## 摘要

**目的:**

旨在量化特定原因死亡和从多个致死原因中选取主要死因的报告变化是如何影响 65 岁或以上的美国人意外伤害死亡率的趋势变化。

**方法:**

我们提取了美国疾病控制和预防中心在线数据库中 1999 年至 2016 年年龄标准化的意外伤害死亡率数据。我们使用归因法来计算两个指标：在所有死亡中使用因特定原因死亡的部分；以及从多个致死原因中选取主要死因的部分。我们采取线性回归的分析方法来探讨这些比例、报告的死亡率以及年龄标准化死亡率随时间推移的变化。

**结果:**

从 1999 年到 2016 年，基于死亡中的多重原因，这一年龄组的特定死因的意外伤害死亡率从 1999 年的 74%（每 10 万人中，185.0 人中有 136.9 人死亡）上升到 2016 年的 85%（每 10 万人中，169.1 人中有 143.0 人死亡）。至于从多个致死原因中选取主要死因的死亡率，除机动车碰撞事故外，其在所有特定意外伤害原因的死亡率中均有所上升。1999 年至 2016 年间，报告的变化导致年龄标准化死亡率稳步上升。每 10 万人中，意外伤害、坠落、机动车辆碰撞事故、窒息、中毒以及火灾或烧伤的死亡率分别上升了 24.2、13.5、2.1、2.3、1.6 以及 0.4 。

**结论:**

随着时间的推移，数据报告的变化影响美国老年人意外伤害死亡率的总体和特定趋势。

## Резюме

**Цель:**

Количественно оценить, каким образом изменения в отчетности с указанием конкретной причины смерти и выбор основополагающей причины из нескольких возможных причин смерти вносят вклад в тенденции смертности от непредумышленных травм у американцев в возрасте от 65 лет и старше.

**Методы:**

Авторы воспользовались данными о смертности от непредумышленных травм, стандартизированными по возрасту, из онлайновых баз данных Центров США по контролю и профилактике заболеваний за период с 1999 по 2016 год. С помощью методов атрибуции были рассчитаны два показателя: доля смертей с определенными кодами среди всех случаев смерти и доля смертей, для которых основополагающая причина выбиралась из ряда возможных причин смерти. Была выполнена линейная регрессия для изучения изменений этих пропорций во времени, а также в составе отчетности по смертности в целом и по смертности с поправкой на возраст.

**Результаты:**

За период с 1999 по 2016 год доля случаев смерти от непредумышленных травм, имеющих определенную причину, выросла в указанной возрастной группе с 74% в 1999 г. (136,9 из 185,0 случая на 100 000 населения) до 85% в 2016 г. (143,0 из 169,1 случая на 100 000 населения) на основании кодов смертных случаев с несколькими причинами. Доля случаев смертности, в которых основополагающую причину смерти выбирали из списка нескольких возможных причин, выросла для всех отдельно рассматриваемых причин гибели от непредумышленных травм, кроме случаев аварий автотранспортных средств. Стандартизированная по возрастному показателю смертность вносила вклад в постепенный рост изменений в отчетности с 1999 по 2016 год. Рост общего количества непредумышленных травм, падений, аварий автотранспортного средства, удушений, отравлений и смертей от пожара или горячего предмета составил соответственно 24,2; 13,5; 2,1; 2,3; 1,6 и 0,4 случая смерти на 100 000 человек.

**Вывод:**

Изменения в отчетности с течением времени влияют на тенденции общей смертности от непредумышленных травм и отдельных конкретных групп внутри этого общего показателя для пожилых американцев.

## Introduction

Proper interpretation of reported injury mortality rates and their changes over time are important for assessing the effects of sociodemographic factors on injury risk, developing injury control and prevention efforts, and prioritizing policy interventions. The underlying cause of death is defined as the disease or injury that initiated the train of events leading directly to death, or the circumstances which produced a fatal injury.^1^ Underlying cause-of-death statistics, from which injury mortality rates are derived, are therefore often used to inform researchers, policy-makers and the public. Most users assume the statistics are valid.

The validity of underlying cause-of-death statistics, however, depends highly on the quality of multiple causes of death listings on death certificates. Two factors important for quality reporting are the correctness and specificity of the data on multiple causes of death and the correct selection of the underlying cause of death from the multiple causes of death.^1^^,^^2^ The correctness and specificity of multiple causes of death is primarily determined by clinical diagnosis and the skill of staff members who are responsible for completing death certificates. This includes both their skill in proper recording of each cause of death and in proper ranking of the sequence of multiple causes of death.^1^^,^^2^ Selection of the underlying cause of death from multiple causes of death is typically made according to the sequence of multiple causes of death on the death certificate, with the first cause in the sequence usually chosen.^2^

Over the last two decades, several efforts have been made to improve mortality reporting in the United States of America. In 1996, an international collaborative effort on automating mortality statistics was implemented in Washington, DC. The aim was to develop automated coding systems that assist in performing causal death coding, editing, selection of underlying cause of death and classification of multiple causes of death based on standardized decision tables.^3^ Since that time, automated computer systems such as the Automated Classification of Medical Entities system have been established, refined and expanded.^4^ The systems were demonstrated to be effective adjuncts to the traditional process of coding and classifying the underlying cause of death.^2^

Starting in 2001, the National Center for Health Statistics (NCHS) has conducted regular training on automated computer systems for staff members who conduct mortality coding and selection, plus substantial curriculum revision for that training.^4^ These efforts appear to have improved the accuracy of data reporting of specific causes of death and selecting underlying cause of death from multiple causes of death in the country.^3^^–^^8^

One specific challenge in coding of injury-related deaths is that data-reporting practices on death certificates vary across age cohorts.^9^ Elderly individuals have complex patterns of comorbidity, and their death may by delayed following a minor injury. For older people, therefore, coders less commonly attribute the underlying cause of death to the specific cause of the unintentional injury.^10^ As an example, three recent publications suggest that changes in data reporting in the United States appear to have had a potentially misleading impact on the increase in deaths from unintentional falls among older Americans.^11^^–^^13^

Despite the evidence about falls, the impact of recent changes in mortality data reporting has not been systematically examined in the United States. We do not know the extent to which these changes might affect trends in mortality, especially for older Americans. We therefore conducted this study to assess the change in two common data reporting practices: (i) cause specificity, and (ii) selection of underlying cause of death from multiple causes of death. We also aimed to quantify the contributions of these practices to reported unintentional injury mortality for Americans older than 64 years from 1999 to 2016.

## Methods

### Data source

We used data from the Wide-ranging OnLine Data for Epidemiologic Research databases^2^ of the United States Centers for Disease Control and Prevention (CDC) web portal. These online databases provide public access to ad hoc queries, summary statistics, maps, charts and data extracts for many health-related data. The databases provide data on the number of deaths, crude death rates, age-standardized death rates and 95% confidence intervals (CI) for death rates based on death certificates for United States residents in the 50 States and the District of Columbia. Each entry for a death contains a single underlying cause of death; up to 20 additional multiple causes of death; and demographic data of the person, including place of residence, age, race, Hispanic or non-Hispanic ethnicity, gender, place of death, time of death and whether an autopsy was performed. Mortality data from the death certificates are either entered by the physician certifying a death, coded by the States and provided to NCHS through the Vital Statistics Cooperative Program; or they are coded by NCHS from copies of the original death certificate provided to NCHS by the State registration offices.^2^^,^^14^ Causes of death are coded according to the International Statistical Classification of Diseases and Related Health Problems, 10th revision (ICD-10).^2^ The age-standardized mortality rates in the databases are calculated by multiplying the age-specific death rate for each age group (classified by 10-year intervals, except for people aged under 1 year and 85 years and over) by the corresponding weight from the specified standard population (the year 2000 projected population). These are summed across all age groups and the results are multiplied by 100 000.^2^

We extracted age-standardized mortality rates, and their standard errors, for deaths coded as unintentional injury for Americans aged 65 years and older from 1999 (the earliest data) to 2016. We extracted data for both underlying cause of death and multiple causes of death. Data extraction was completed from January to April 2018. Based on the injury mortality matrix for ICD-10 recommended by the CDC,^15^ we classified the external causes of unintentional injury as: all unintentional injury; unspecified unintentional injury; and five major causes including fall, motor vehicle crash, suffocation, poisoning and fire or hot object (Box 1).

Box 1 **ICD-10 codes used in the study of unintentional injury mortality attributed to changes in data reporting for Americans aged 65 years and older, 1999–2016**

1. All unintentional injury: codes V01–X59, Y85–Y86

2. Unspecified unintentional injury: code X59

3. Fall: codes W00–W19

4. Motor vehicle crash: codes V02–V04, V09.0, V09.2, V12–V14, V19.0–V19.2, V19.4–V19.6, V20–V79, V80.3–V80.5, V81.0–V81.1, V82.0–V82.1, V83–V86, V87.0–V87.8, V88.0–V88.8, V89.0, V89.2

5. Suffocation: codes W75–W84

6. Poisoning: codes X40–X49

7. Fire or hot object: codes X00–X19

ICD-10: International Statistical Classification of Diseases and Related Health Problems 10th revision.Source: External cause of injury mortality matrix for ICD-10, 2002.^15^

### Attribution model

During the reporting process of an injury-related death, the determination of the underlying cause of death from multiple causes of death can be divided into two consecutive and independent steps.

The first step is the reporting of multiple causes of death. To assess diagnoses, record, classify and sequence multiple causes of death, coders usually rely on external criteria, which are based on autopsy reports, physician review panels and querying of complete medical records (i.e. contacting certifiers for clarification).^7^^,^^8^ When detailed medical documents are lacking, deaths are typically recorded by certifiers as unspecified.^2^ Following previous studies,^5^^–^^8^ we obtained the unintentional injury mortality with specified causes recoded among multiple causes of death (*MCD*) codes and calculated this as the proportion of all-cause unintentional injury mortality (*A*):


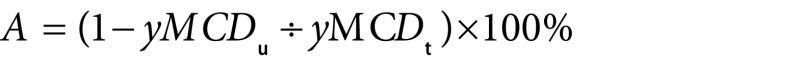
(1)

where *y* represents mortality, *u* represents unspecified-unintentional-injury and *t* all-unintentional-injury. This indictor approximately measures the specificity of injury causes; an increase or decrease in the proportion suggests improvement or deterioration in cause-specific reporting, respectively.^5^^,^^7^

The second step is selecting the underlying cause of death from multiple causes of death. How coders do this is determined by the sequence of conditions on the certificate, the provisions of the ICD-10 and the associated selection rules and modifications.^2^^,^^16^ According to the ICD rules, the sequencing of multiple causes of death determines the choice of the underlying cause of death in most cases.^14^ Therefore, the process reflects the quality of the sequencing of multiple causes, which depends on the knowledge, skill and diligence of the coders. We obtained the unintentional injury mortality recorded in underlying cause-of-death (*UCD*) codes and calculated this as a proportion of unintentional injury mortality recorded in multiple causes of death (*MCD*) codes (*B*): 


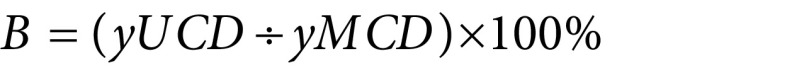
(2)

This indictor roughly reflects the quality of sequencing of multiple causes of death; a higher proportion indicates better quality coding.

We then performed two steps to estimate the contribution of changes in data reporting (of cause specificity and selection of underlying cause of death from multiple causes of death) to mortality data coded as unintentional injury. First, we adopted the widely used strategy of age-standardization to calculate adjusted unintentional injury mortality by assuming that data reporting remained unchanged from 1999 to 2016. The adjusted mortality (*aM*) from the underlying cause of death (*UCD*) for a specific cause *i* in a certain year *m* (mortality *UCD_i_*_,_*_m_*) can be calculated as:


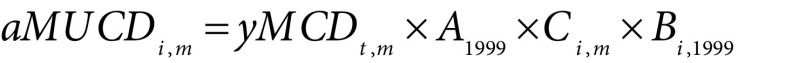
(3)

In this equation *i* means specific injury cause, with *i* = 1, …, 5, representing five major causes of unintentional injury (fall, motor vehicle crash, suffocation, poisoning and fire or hot object, respectively). *m* denotes the year, with *m* = 1999, …, 2016. *A*_1999_ denotes the proportion of unintentional injury mortality from multiple causes of death (mortality *MCD_t_*) with specific ICD-10 codes out of all-cause unintentional injury mortality from multiple causes of death in 1999. *C_i_*_,_*_m_* represents the proportion of injury mortality recorded as the cause *i* in injury mortality with specific ICD-10 codes in the year *m*. This proportion reflects the combined impact of risk factors, injury control efforts and quality of data reporting (typically, misclassification across causes).^17^^,^^18^ Due to a lack of relevant data, we could not separate the effects of data reporting from that of risk factors and injury control efforts, and thus we used the proportion in year *m* here. *B_i_*_,1999_ means the proportion of underlying cause of death selected from multiple causes of death for a given specific cause *i* in 1999.

Second, we calculated the difference between adjusted mortality *UCD_i_*_,_*_m_* and reported mortality *UCD_i_*_,_*_m_*. The difference reflects the contribution of changes in data reporting to unintentional injury mortality for a specific cause *i* in year *m* compared with year 1999.

### Statistical analysis

We conducted the analyses in three steps. First, we plotted line graphs to demonstrate changes in the proportion of unintentional injury mortality with (i) specific external causes (i.e. cause specificity) and (ii) proportion of underlying cause of death selected out of multiple causes of death over years. The 95% CI of the two proportions were estimated using normal approximation due to large sample size. Second, we plotted stacked bar charts to show unintentional injury mortality attributed to data reporting changes from 1999 to 2016. Finally, we made a linear regression to examine changes in the proportion of unintentional injury mortality with specific external causes; selection proportion of underlying cause of death from multiple causes of death; reported and adjusted unintentional injury mortality; and unintentional injury mortality by data reporting changes from 1999 to 2016. We calculated the robust standard errors to provide valid inferences even under model misspecification.^19^ Because differences between robust and classical standard errors of regression coefficients were extremely small (from 0.00001 to 0.01944), we decided to use classical methods to estimate the standard errors of regression coefficients (*β*). We calculated percentage changes in injury mortality between 1999 and 2016 as: 


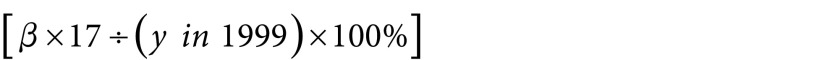
(4)

We performed all data analyses using Stata, version 12.1 software (Stata Corp., College Station, United States). We considered differences to be statistically significant in two-tailed tests if *P*-values were less than 0.05.

### Ethical statement

This study used anonymous open-access data and did not involve personal information from individuals. Our use of data complied strictly with the terms of the data use restrictions of the CDC online databases. The research proposal was approved by the ethics committee of Xiangya School of Public Health, Changsha, China (no. XYGW-2016–28).

## Results

**Fig. 1:**
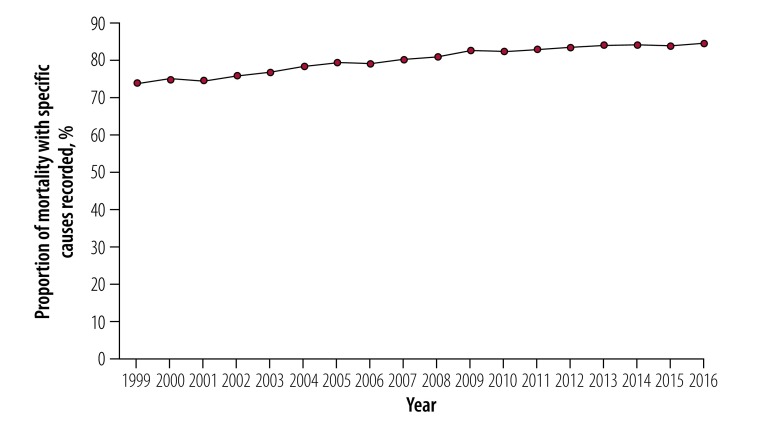
Proportion of mortality with specific causes recorded out of all unintentional injury in multiple causes of death codes for Americans aged 65 years and older, 1999–2016

Notes: We extracted age-standardized mortality data from of the United States Centers for Disease Control and Prevention online databases;^2^ causes of death are recorded by International Statistical Classification of Diseases and Related Health Problems (10th revision) codes.^16^

**Fig. 2:**
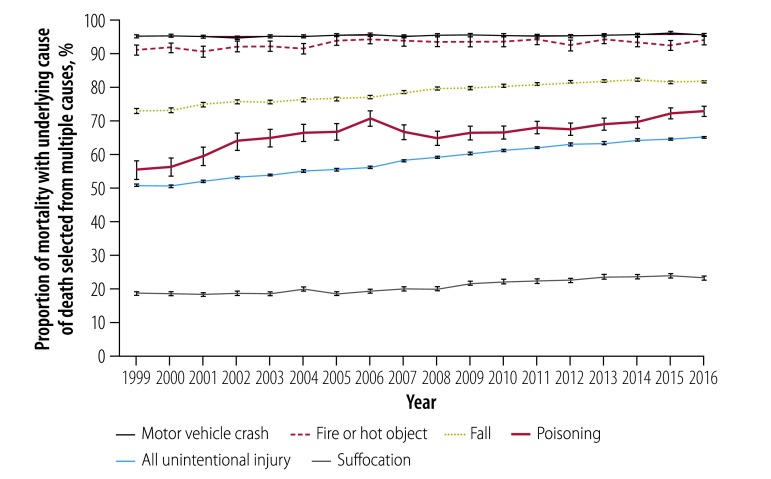
Proportion of mortality from unintentional injury selected as underlying cause of death among multiple causes of death codes for Americans aged 65 years and older, 1999–2016

Notes: The error bars show 95% confidence interval of the proportion. We extracted age-standardized mortality data from of the United States Centers for Disease Control and Prevention online databases;^2^ causes of death are recorded by International Statistical Classification of Diseases and Related Health Problems (10th revision) codes.^16^

The total population aged 65 years and older was 34 797 841 in 1999 and 49 244 195 in 2016. The age-standardized mortality from all-cause unintentional injuries was 185.0 per 100 000 persons in 1999 and 163.9 per 100 000 in 2016, based on multiple causes of death codes. We found that the proportion of unintentional injury mortality with specific external causes recorded increased significantly from 74% in 1999 (136.9 per 100 000) to 85% in 2016 (143.0 per 100 000; *b* = 0.0067, *P* < 0.01; Table 1; Fig. 1). The proportion of underlying cause of death selected from multiple causes of death also rose significantly between 1999 and 2016 from 51% (93.6 per 100 000) to 65% (109.8 per 100 000). Increases in the selection proportion were found for specific causes (fall: 73% versus 81%; suffocation: 18% versus 23%; poisoning: 55% versus 73%; fire or hot object: 91% versus 94%), except for motor vehicle crash (95% versus 95%; Fig. 2). 

**Table 1:** Specificity of injury cause and quality of sequencing of multiple causes of death from unintentional injury among Americans aged 65 years and older, 1999–2016

Year	Population	Age-standardized mortality per 100 000 persons				
		All unintentional injury	Unspecified unintentional injury	Cause specificity indicator, %	Unintentional injury selected as underlying cause	Cause selection indicator, %
1999	34 797 841	185.0	48.1	74	93.6	51
2000	34 991 753	177.3	44.5	75	89.2	50
2001	35 290 291	178.6	45.4	75	92.6	52
2002	35 522 207	178.0	42.8	76	94.4	53
2003	35 863 529	177.2	41.3	77	95.0	54
2004	36 203 319	174.4	37.8	78	95.8	55
2005	36 649 798	178.7	36.7	79	98.7	55
2006	37 164 107	172.8	36.1	79	96.8	56
2007	37 825 711	170.8	34.0	80	98.9	58
2008	38 777 621	169.0	32.2	81	99.5	59
2009	39 623 175	161.1	28.0	83	96.9	60
2010	40 267 984	165.0	29.1	82	100.6	61
2011	41 394 141	165.3	28.3	83	102.2	62
2012	43 145 356	163.2	27.1	83	102.6	63
2013	44 704 074	162.2	26.0	84	102.6	63
2014	46 243 211	163.9	26.1	84	105.0	64
2015	47 760 852	169.3	27.2	84	108.9	64
2016	49 244 195	169.1	26.1	85	109.8	65

Notes: We extracted age-standardized mortality data from of the United States Centers for Disease Control and Prevention online databases;^2^ causes of death are recorded by International Statistical Classification of Diseases and Related Health Problems (10th revision) codes.^16^ All unintentional injury mortality is the total mortality from all causes recorded in multiple causes of death codes. The cause specificity indicator is the proportion of unintentional injury mortality with specific causes recorded out of all unintentional injury mortality recorded in multiple causes of death codes. The cause selection indicator is the proportion of unintentional injury mortality recorded in underlying cause of death codes selected from unintentional injury mortality recorded in multiple causes of death codes.

**Fig. 3:**
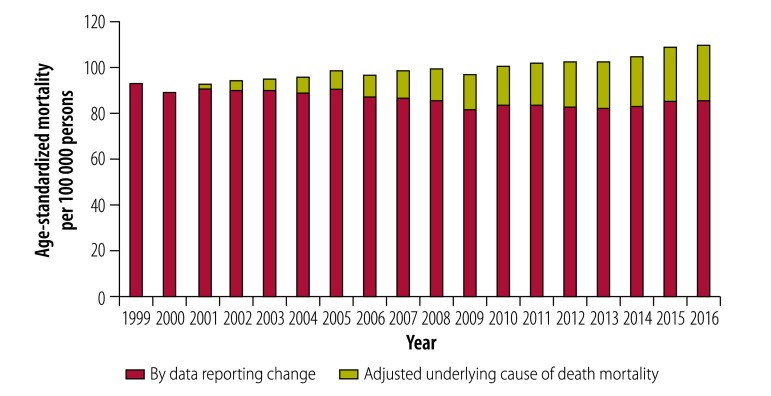
Age-standardized mortality by adjusted underlying cause of death mortality and by data reporting change for Americans aged 65 years and older, 1999–2016: all unintentional injury

Notes: We defined adjusted underlying cause of death mortality as mortality from underlying cause of death calculated assuming that data reporting (cause specificity and selection of underlying cause of death from multiple causes of death) remained unchanged from 1999 to 2016. We defined data reporting change as the difference between adjusted underlying cause of death mortality and reported underlying cause of death mortality.

Age-standardized mortality attributed to data reporting changes (namely, improvement in cause specificity and selection of underlying cause of death based on multiple causes of death) rose for overall and specific injury deaths from 1999 to 2016 (*P* < 0.05). Compared with 1999, data reporting changes contributed 24.2 per 100 000 persons to reported mortality rates based on underlying cause of death in 2016 for all unintentional injuries (Fig. 3). Corresponding figures for the five major causes of injury were 13.5 per 100 000 persons for fall, 2.1 for motor vehicle crash, 2.3 for suffocation, 1.6 for poisoning and 0.4, for fire or hot object due to data reporting changes.

**Fig. 4:**
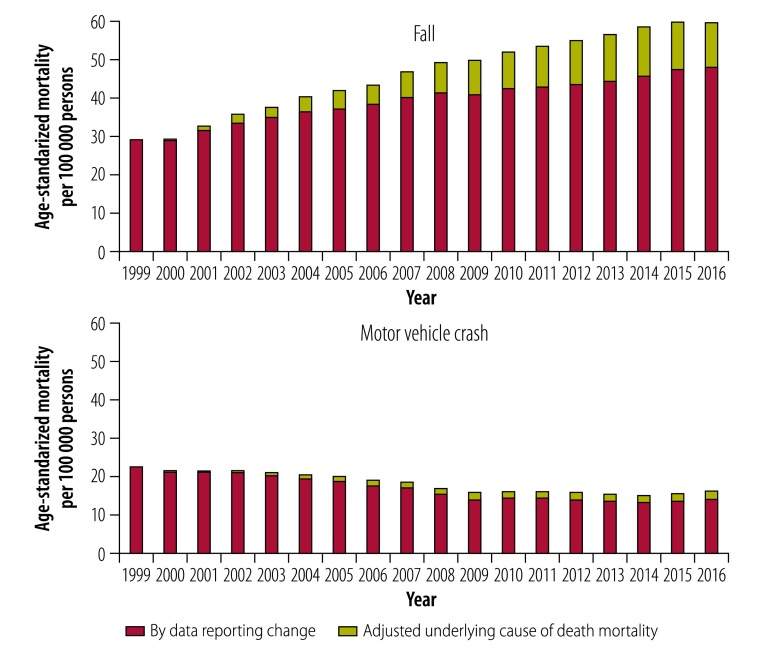
Age-standardized mortality by adjusted underlying cause of death mortality and by data reporting change for Americans aged 65 years and older, 1999–2016: unintentional injury from fall and motor vehicle crash

**Fig. 5:**
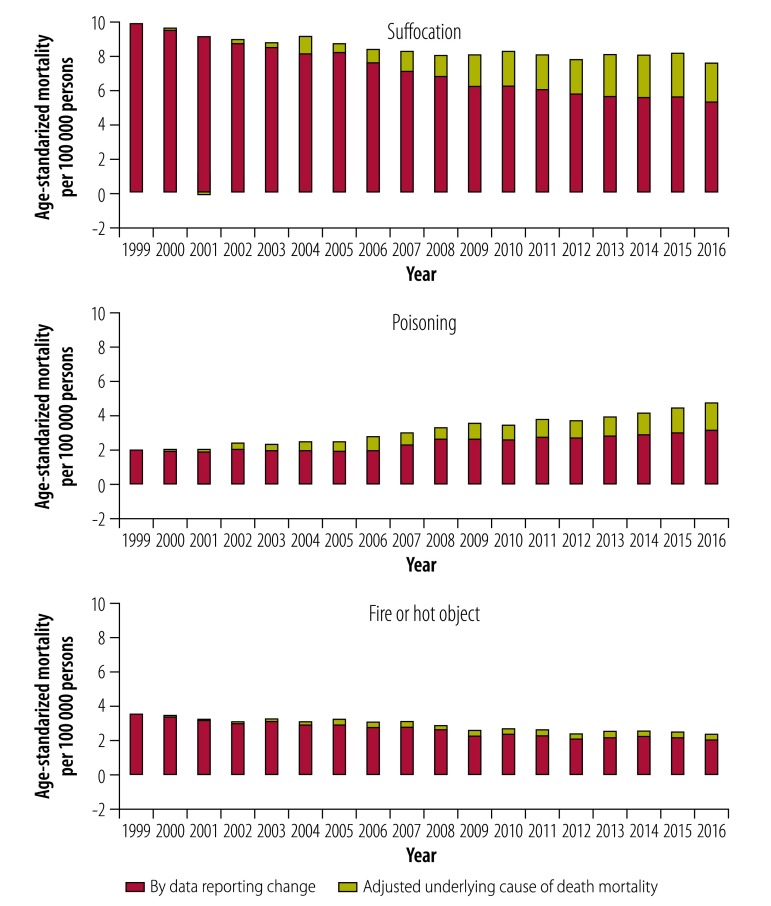
Age-standardized mortality by adjusted underlying cause of death mortality and by data reporting change for Americans aged 65 years and older, 1999–2016: unintentional injury from suffocation, poisoning and fire or hot object

Trend analysis showed data reporting changes significantly distorted and even reversed trends in overall and cause-specific mortality from underlying cause of death. The percentage change in mortality between 1999 and 2016 greatly differed between reported versus adjusted unintentional injury mortality: 18% versus −10% for overall unintentional injury (Table 2 and Fig. 3). Changes for the five major causes of injury were: 113% versus 63% for fall; −35% versus −44% for motor vehicle crash (Fig. 4); −18% versus −48% for suffocation; 134% versus 64% for poisoning; and −32% versus −42% for fire or hot object (Table 2 and Fig. 5).

**Table 2:** Percentage change in adjusted and reported mortality for underlying cause of death among Americans aged 65 years and older between 1999 and 2016: overall and cause-specific unintentional injuries

Variable	Age-standardized mortality per 100 000 persons					
	Reported			Adjusted^a^		
	1999	2016	% change (95% CI)	1999	2016	% change (95% CI)
**All unintentional injury**	93.6	109.8	18 (15 to 21)	93.6	85.6	−10 (−13 to −6)
**Selected specific cause**						
Fall	29.4	61.6	113 (107 to 119)	29.4	48.1	63 (57 to 68)
Motor vehicle crash	22.4	16.3	−35 (−42 to −29)	22.4	14.2	−44 (−52 to −37)
Suffocation	9.9	7.6	−18 (−23 to −14)	9.9	5.3	−48 (−53 to −43)
Poisoning	2.0	4.8	134 (123 to 146)	2.0	3.2	64 (52 to 76)
Fire or hot object	3.6	2.4	−32 (−37 to −26)	3.6	2.0	−42 (−48 to −36)

CI: confidence interval.

^a^ Adjusted mortality from the underlying cause of death was calculated by assuming that data reporting (cause specificity and selection of underlying cause of death from multiple causes of death) remained unchanged from 1999 to 2016.

Notes: We extracted age-standardized mortality data from of the United States Centers for Disease Control and Prevention online databases;^2^ causes of death are recorded by International Statistical Classification of Diseases and Related Health Problems (10th revision) codes.^16^ Percentage change in mortality between 1999 and 2016 was calculated as: [regression coefficient × 17 ÷ (mortality in 1999) × 100%]. The total population aged 65+ years in the 50 States and District of Columbia of the United States of America was 34 797 841 in 1999 and 49 244 195 in 2016.

## Discussion

### Primary findings

Using the latest mortality data, we assessed two changes in data reporting – cause specificity and selection of underlying cause of death from multiple causes of death – and quantified their impact on unintentional injury mortality reports for Americans aged 65 years and older. The results reveal that a gradual increase in recording specificity of cause of death and an improvement in selecting the underlying cause of death from 1999 to 2016 caused a growing contribution to overall and specific unintentional injury mortality over time. These reporting changes altered published trends in unintentional injury mortality for older Americans, overestimating increases in mortality from overall unintentional injuries, fall and poisoning, and underestimating decreases in mortality from motor vehicle crash, suffocation and fire or hot object to some extent.

### Validity of attribution method

The attribution method we propose is based on the built knowledge on roughly measuring cause specificity and selection of underlying cause of death from multiple causes of death through two surrogate indicators (cause specificity and selection of underlying cause of death from multiple causes of death). The two indicators are especially valuable when external and complete information for quality assessment is unavailable or inaccessible.

The proportion of mortality with specific ICD-10 codes out of all mortality approximately reflects the specificity of multiple causes of death, so it measures the quality of codes and sufficiency of information on the death certificates.^5^^–^^8^ The proportion of underlying cause of death selected from multiple causes of death depends on the sequencing of multiple causes of death in most cases and follows the selection rules of underlying cause of death from the ICD.^14^ The sequencing of multiple causes of death further relies on the knowledge, skill and diligence of coders. Thus, the selection proportion of underlying cause of death from multiple causes of death approximately reflects data reporting quality;^20^^,^^21^ this strategy has been used previously.^22^

### Interpretation of findings

This study replicates previous findings suggesting that reporting of specific causes of death for unintentional injury among older adults has improved. This change in reporting has had an impact on reported cause-specific unintentional injury mortality in the United States.^6^^–^^8^ Our study extends those findings in several ways, by visualizing the improvements in selection of underlying cause of death from multiple causes of death. We also describe a strategy to quantify the combined contribution of changes in cause specificity and selection of underlying cause of death from multiple causes of death to the reported mortality from multiple causes of death from 1999 to 2016.

The data reporting improvements we observed are likely to be a result of continuous and enhanced efforts by federal, state and local governments in the United States over the past three decades. These efforts include improved quality of training and practice for coding and selection^23^^–^^26^ and the development of automated coding and classification systems using computer procedures that implement standardized selection rules consistent with ICD-10 modifications.^3^^,^^4^^,^^14^^,^^16^^,^^27^ For example, New York city created an eLearning instructional course on cause-of-death reporting in 2008 and mandated this course for users of the electronic death registration system in 2010.^3^^,^^4^ Empirical research demonstrated these changes were successful in teaching medical professionals to report cause of death accurately.^23^^,^^24^

### Implications

Our findings have several implications. First, we documented that reporting of unintentional injury mortality among older Americans has gradually improved, yielding significant effects on reported mortality from the underlying cause of death. For this reason, data reporting changes must be considered when using mortality from underlying cause of death to examine the extent of injury mortality and the effectiveness of interventions over time, as well as to develop and evaluate national and local health initiatives such as Healthy People 2020 and 2030.^28^ In practice, two strategies can be used to address data reporting changes. The first strategy is to completely and routinely assess data reporting quality (including cause specificity, selection of the underlying cause of death from multiple causes of death, and completeness and misclassification). Its impact on reported injury mortality from the underlying cause of death can then be adjusted at federal, state and local levels before releasing data. An alternative strategy is to provide users with warnings that the mortality rates of specific subgroups or certain causes might be heavily affected by changes in data reporting. The first option requires considerable resources, but may be more effective; the second option may be more feasible in many lower-resource environments.

Second, our findings reinforce the vital role that data quality plays in evidence-based research and decision-making. Although our findings were based on American data, the results are relevant globally, as many other countries undergo regular data reporting changes. For example, Australia, France, Italy, the Netherlands and Sweden, and have successively introduced and converted to automated computer systems over the past two decades.^4^ These transitions have been shown to yield data changes (e.g. in pneumonia deaths in Australia after the introduction of automated computer systems).^29^ Similarly, a recent analysis of 195 countries reported that 26 countries had improved data consistency between health and non-health data for road traffic mortality from 1985 to 2013, and nine countries had decreased data consistency.^30^

Last, although the changes in data reporting we report here yield a growing impact on overall and specific unintentional injury mortality, the actual mortality caused by unintentional injury remains high among older Americans. Continued efforts to develop and disseminate effective prevention strategies are needed in addition to efforts to improve data quality.

### Study limitations

These findings should be viewed in the light of some limitations of the research. First, although we quantitatively attributed changes in specificity and selection of reporting the underlying cause of unintentional injury deaths from multiple causes of death, such attribution assumes all other factors remain unchanged over the study period. In reality, data reporting changes are likely interacting with other factors that we did not consider. Second, due to lack of detailed data, we could not quantify the contribution of other attributes of data reporting (e.g. completeness of reporting and misclassification across diseases and injuries).^31^ Further studies are needed to systematically assess recent changes in data reporting and their impact on injury mortality statistics.

### Conclusions

Injury mortality data reporting in the United States has changed significantly over 1999–2016 in both the reporting of the specific causes of death and in the selection of underlying cause of death from multiple causes of death. These changes created an apparent contribution to increasing unintentional injury mortality for Americans aged 65 years and older and significantly altered trends in overall and specific unintentional injury mortality. The potential impact of changes in data reporting should be considered by researchers, policy-makers and other stakeholders who interpret and use the data.

## References

[1] Kircher T, Anderson RE. Cause of death. Proper completion of the death certificate. JAMA. 1987 7 17;258(3):349–52. 10.1001/jama.1987.03400030065033 3599328

[2] CDC WONDER online databases [internet]. Atlanta: Centers for Disease Control and Prevention, US Department of Health & Human Services; 2018. Available from: http://wonder.cdc.gov/ [cited 2018 Mar 23].

[3] Peters K, editor. Proceedings of the International Collaborative Effort on Automating Mortality Statistics, Volume 1. DHHS Publication No. (PHS) 99-1252. Hyattsville: National Center for Health Statistics, Centers for Disease Control and Prevention, US Department of Health & Human Services; 1999. Available from: https://www.cdc.gov/nchs/data/misc/ice99_1.pdf [cited 2018 Mar 25].

[4] Minino AM, Rosenberg Harry M, editors. Proceedings of the International Collaborative Effort on Automating Mortality Statistics, Volume 2. Hyattsville: National Center for Health Statistics, Centers for Disease Control and Prevention, US Department of Health & Human Services; 2001. Available from: https://www.cdc.gov/nchs/data/misc/ice01_acc.pdf [cited 2018 Mar 25].

[5] Lu TH, Walker S, Anderson RN, McKenzie K, Bjorkenstam C, Hou WH. Proportion of injury deaths with unspecified external cause codes: a comparison of Australia, Sweden, Taiwan and the US. Inj Prev. 2007 8;13(4):276–81. 10.1136/ip.2006.012930 17686940 PMC2598354

[6] Kharrazi RJ, Nash D, Mielenz TJ. Increasing trend of fatal falls in older adults in the United States, 1992 to 2005: coding practice or reporting quality? J Am Geriatr Soc. 2015 9;63(9):1913–7. 10.1111/jgs.13591 26200220

[7] Hu G, Mamady K. Impact of changes in specificity of data recording on cause-specific injury mortality in the United States, 1999–2010. BMC Public Health. 2014 9 27;14(1):1010. 10.1186/1471-2458-14-1010 25262245 PMC4246427

[8] Cheng X, Wu Y, Yao J, Schwebel DC, Hu G. Mortality from unspecified unintentional injury among individuals aged 65 years and older by U.S. state, 1999–2013. Int J Environ Res Public Health. 2016 7 27;13(8):763. 10.3390/ijerph13080763 27472356 PMC4997449

[9] Dijkhuis H, Zwerling C, Parrish G, Bennett T, Kemper HC. Medical examiner data in injury surveillance: a comparison with death certificates. Am J Epidemiol. 1994 3 15;139(6):637–43. 10.1093/oxfordjournals.aje.a117053 8172175

[10] Baker SP, O’Neill B, Haddon W Jr, Long WB. The injury severity score: a method for describing patients with multiple injuries and evaluating emergency care. J Trauma. 1974 3;14(3):187–96. 10.1097/00005373-197403000-00001 4814394

[11] Hu G, Baker SP. Recent increases in fatal and non-fatal injury among people aged 65 years and over in the USA. Inj Prev. 2010 2;16(1):26–30. 10.1136/ip.2009.023481 20179032

[12] Hu G, Baker SP. An explanation for the recent increase in the fall death rate among older Americans: a subgroup analysis. Public Health Rep. 2012 May-Jun;127(3):275–81. 10.1177/003335491212700307 22547858 PMC3314071

[13] Alamgir H, Muazzam S, Nasrullah M. Unintentional falls mortality among elderly in the United States: time for action. Injury. 2012 12;43(12):2065–71. 10.1016/j.injury.2011.12.001 22265137

[14] National Center for Health Statistics instruction manual. Part 2a: instructions for classifying the underlying cause of death. Hyattsville: National Center for Health Statistics, Centers for Disease Control and Prevention, US Department of Health & Human Services; 2008. Available from: http://www.cdc.gov/nchs/data/dvs/2a2008Final.pdf [cited 2018 Apr 5].

[15] External cause of injury mortality matrix for ICD-10. Atlanta: Centers for Disease Control and Prevention, US Department of Health & Human Services; 2002. Available from: https://www.cdc.gov/nchs/data/ice/icd10_transcode.pdf [cited 2018 Mar 21].

[16] International statistical classification of diseases and related health problems, 10th revision. Volume 2: instruction manual. 5th ed. Geneva: World Health Organization; 2016. Available from: http://apps.who.int/classifications/icd10/browse/Content/statichtml/ICD10Volume2_en_2016.pdf [cited Feb 23 2018].

[17] Improving the quality and use of birth, death and cause-of-death information: guidance for a standards-based review of country practices. Geneva: World Health Organization; 2010. Available from: http://apps.who.int/iris/bitstream/handle/10665/44274/9789241547970_eng.pdf;jsessionid=CE20FB8F3E9705CF1F17849A0926D22F?sequence=1 [cited 2018 Mar 24].

[18] Lahti RA, Penttilä A. Cause-of-death query in validation of death certification by expert panel; effects on mortality statistics in Finland, 1995. Forensic Sci Int. 2003 1 28;131(2-3):113–24. 10.1016/S0379-0738(02)00418-8 12590049

[19] Huber PJ. Robust statistics. International encyclopedia of statistical science. Berlin: Springer; 2011.

[20] Lu TH, Hsiao A, Chang PC, Chao YC, Hsu CC, Peng HC, et al. Counting injury deaths: a comparison of two definitions and two countries. Inj Prev. 2015 4;21 e1:e127–32. 10.1136/injuryprev-2013-040974 24345723

[21] Lu TH, Lin JJ. Using multiple-cause-of-death data as a complement of underlying-cause-of-death data in examining mortality differences in psychiatric disorders between countries. Soc Psychiatry Psychiatr Epidemiol. 2010 8;45(8):837–42. 10.1007/s00127-009-0127-0 19727532

[22] Goldberger N, Applbaum Y, Meron J, Haklai Z. High Israeli mortality rates from diabetes and renal failure: can international comparison of multiple causes of death reflect differences in choice of underlying cause? Isr J Health Policy Res. 2015 10 1;4(1):31. 10.1186/s13584-015-0027-6 26430506 PMC4590706

[23] Hemans-Henry C, Greene CM, Koppaka R. Integrating public health–oriented e-learning into graduate medical education. Am J Public Health. 2012 6;102(S3) Suppl 3:S353–6. 10.2105/AJPH.2012.300669 22690971 PMC3478079

[24] Madsen A, Begier E. Improving quality of cause-of-death reporting in New York City. Prev Chronic Dis. 2013 7 18;10:130227. 10.5888/pcd10.130227 23866162 PMC3716335

[25] Madsen A, Thihalolipavan S, Maduro G, Zimmerman R, Koppaka R, Li W, et al. An intervention to improve cause-of-death reporting in New York City hospitals, 2009-2010. Prev Chronic Dis. 2012;9:120071. 10.5888/pcd9.120071 23078668 PMC3477897

[26] Al-Samarrai T, Madsen A, Zimmerman R, Maduro G, Li W, Greene C, et al. Impact of a hospital-level intervention to reduce heart disease overreporting on leading causes of death. Prev Chronic Dis. 2013 5 16;10:120210. 10.5888/pcd10.120210 23680506 PMC3667027

[27] Stanfill MH, Williams M, Fenton SH, Jenders RA, Hersh WR. A systematic literature review of automated clinical coding and classification systems. J Am Med Inform Assoc. 2010 Nov-Dec;17(6):646–51. 10.1136/jamia.2009.001024 20962126 PMC3000748

[28] Healthy People 2020 [internet]. Washington: Office of Disease Prevention and Health Promotion; 2018. Available from: https://www.healthypeople.gov/ [cited 2018 Apr 7].

[29] McKenzie K, Walker S, Tong S. Assessment of the impact of the change from manual to automated coding on mortality statistics in Australia. Health Inf Manag. 2002;30(3):1–11. 19468137

[30] Huang H, Yin Q, Schwebel DC, Ning P, Hu G. Availability and consistency of health and non-health data for road traffic fatality: Analysis of data from 195 countries, 1985–2013. Accid Anal Prev. 2017 11;108:220–6. 10.1016/j.aap.2017.08.033 28915503

[31] Birkhead GS, Klompas M, Shah NR. Uses of electronic health records for public health surveillance to advance public health. Annu Rev Public Health. 2015 3 18;36(1):345–59. 10.1146/annurev-publhealth-031914-122747 25581157

